# A novel immune cell signature for predicting osteosarcoma prognosis and guiding therapy

**DOI:** 10.3389/fimmu.2022.1017120

**Published:** 2022-09-14

**Authors:** Runsang Pan, Feng Pan, Zhirui Zeng, Shan Lei, Yan Yang, Yushi Yang, Chujiao Hu, Houping Chen, Xiaobin Tian

**Affiliations:** ^1^ School of Basic Medicine, Guizhou Medical University, Guiyang, China; ^2^ Department of Bone and Joint Surgery, Guizhou Orthopedics Hospital, Guiyang, China; ^3^ Transformation Engineering Research Center of Chronic Disease Diagnosis and Treatment, Guizhou Medical University, Guiyang, China; ^4^ Department of Pathology, Affiliated Hospital of Guizhou Medical University, Guiyang, China; ^5^ State Key Laboratory of Functions and Applications of Medicinal Plants, Guizhou Medical University, Guiyang, China; ^6^ Guizhou Provincial Engineering Technology Research Center for Chemical Drug R&D, Guizhou Medical University, Guiyang, China; ^7^ Department of Orthopedics, Guiyang Maternal and Child Health-Care Hospital, Guiyang, China; ^8^ Department of Orthopedics, The Affiliated Hospital of Guizhou Medical University, Guiyang, China

**Keywords:** immune cell, signature, osteosarcoma, prognosis, riskscore

## Abstract

Dysregulation of immune cell infiltration in the tumor microenvironment contributes to the progression of osteosarcoma (OS). In the present study, we explored genes related to immune cell infiltration and constructed a risk model to predict the prognosis of and guide therapeutic strategies for OS. The gene expression profile of OS was obtained from TARGET and Gene Expression Omnibus, which were set as the discovery and verification cohorts. CIBERSORT and Kaplan survival analyses were used to analyze the effects of immune cells on the overall survival rates of OS in the discovery cohort. Differentially expressed gene (DEG) analysis and protein–protein interaction (PPI) networks were used to analyze genes associated with immune cell infiltration. Cox regression analysis was used to select key genes to construct a risk model that classified OS tissues into high- and low-risk groups. The prognostic value of the risk model for survival and metastasis was analyzed by Kaplan–Meier survival analyses, receiver operating characteristic curves, and immunohistochemical experiments. Immunological characteristics and response effects of immune checkpoint blockade (ICB) therapy in OS tissues were analyzed using the ESTIMATE and Tumor Immune Dysfunction and Exclusion algorithms, while sensitivity for both targeted and chemotherapy drugs was analyzed using the OncoPredict algorithm. It was demonstrated that the high infiltration of resting dendritic cells in OS tissues was associated with poor prognosis. A total of 225 DEGs were found between the high- and low-infiltration groups of OS tissues, while 94 genes interacted with others. Through COX analyses, among these 94 genes, four genes (including AOC3, CDK6, COL22A1, and RNASE6) were used to construct a risk model. This risk model showed a remarkable prognostic value for survival rates and metastasis in both the discovery and verification cohorts. Even though a high microsatellite instability score was observed in the high-risk group, the ICB response in the high-risk group was poor. Furthermore, using OncoPredict, we found that the high-risk group OS tissues were resistant to seven drugs and sensitive to 25 drugs. Therefore, our study indicates that the resting dendritic cell signature constructed by AOC3, CDK6, COL22A1, and RNASE6 may contribute to predicting osteosarcoma prognosis and thus therapy guidance.

## Introduction

As a primary malignant bone tumor, osteosarcoma (OS) is the leading cause of cancer-related deaths among children and adolescents (1). Currently, surgery and chemotherapy are the primary treatments for OS. Over the past 30 years, the 5-year survival rate of OS has increased to 70%. However, patients with OS still have a poor prognosis due to drug resistance, metastasis, or recurrence (2, 3). Therefore, an urgent need to identify novel biomarkers for OS that may contribute to therapy practices is apparent.

Previous studies have indicated that the dysregulation of immune cells plays a key role in the malignant activity of osteosarcoma, which also includes metastasis and drug resistance (4, 5). Sun et al. demonstrated lower infiltration of CD8-positive T cells in OS tissues and induced OS cell proliferation (6). Shao et al. demonstrated that M2 macrophages are enriched in primary osteosarcoma tissues, thus activating cancer stem cells in osteosarcoma tissues and inducing drug resistance (7). Therefore, immunotherapy (including adoptive cell therapy, vaccination, and checkpoint inhibitors) has become increasingly popular for cancer therapy in recent years (8). Anti-programmed cell death 1 (PD1) and anti-programmed cell death 1 ligand 1 (PDL1) blockade therapies have shown encouraging results in various preclinical studies (9, 10). However, differing from the success of preclinical studies, a randomized clinical trial conducted by Tawbi et al. in 2017 showed that only 5% of patients with OS had an objective response to pembrolizumab—a PD1 antibody. The authors indicated that different patients with OS have different immune microenvironment characteristics and, therefore, have different responses to immunotherapy (11). Hence, studying the immunological characteristics of OS tissues may contribute to improving immunotherapy efficiency.

In the present study, we aimed to explore genes associated with immune cell infiltration and constructed a risk model to predict the prognosis of OS and thus guide therapeutic strategies for OS. We found that high levels of resting dendritic cells were associated with poorer prognoses in OS, and the risk model (constructed using resting dendritic cell-associated genes) may have remarkable value in predicting OS prognosis and guiding therapy.

## Materials and methods

### Data acquisition and preprocessing

Gene expression in OS tissues was acquired from the Therapeutically Applicable Research to Generate Effective Treatments (TARGET; https://ocg.cancer.gov/programs/target) database and Gene Expression Omnibus (GEO; accession number: GSE21257; https://www.ncbi.nlm.nih.gov/gds). For the gene expression profile in TARGET, a total of 85 tissues provided by patients had fully equipped clinical messages, including that of age, sex, and metastasis status. The gene expression of GSE21257 was supplied by Buddingh et al. (12), and 53 tissues provided by patients had fully equipped clinical messages. Prior to the analyses, we translated the probe name into gene symbols and performed batch normalization and centralization.

### Immune cell analysis

CIBERSORT is an R package that calculates cell fractions from bulk tissue gene expression profiles (13). In the present study, we used CIBERSORT to calculate the number of 22 immune cells in OS. The relationship between immune cells and survival rates of patients with OS was analyzed using Kaplan–Meier survival analyses (log-rank), and p < 0.05 was set as the threshold of significance.

### DEG analysis

OS tissues were divided into high- and low-infiltration groups, according to the median level. Differentially expressed genes (DEGs), between the high- and low-infiltration groups, were analyzed using the Limma package (version 3.15), while p < 0.05, and | log fold change (FC)| ≥1 were set as thresholds to select DEGs.

### Protein–protein interaction (PPI) network construction and enrichment analysis

DEGs were imported into STRING (https://cn.string-db.org/), with a reliability>0.4, to establish an initial network. In this network, genes with no interactions were removed. The adjusted initial network was visualized using the Cytoscape software (version 3.6.1). Genes in the network were subjected to enrichment analysis. Analyses of biological process (BP), molecular function (MF), and KEGG pathway enrichment were performed using the DAVID database (https://david.ncifcrf.gov/tools.jsp). Terms with a p value of <0.05 were regarded as significant, and the top five terms were visualized.

### Construction and verification of the risk model

Gene expression and survival data of patients with OS were imported and used to conduct a univariate COX analysis. Then, survival-associated genes (p < 0.05) were subjected to least absolute shrinkage and selection operator (LASSO) COX analysis to select more important survival-associated genes, by adding appropriate penalties (lambda). Finally (utilizing the Akaike information criterion), an optimal prognostic risk model was built, using a multivariate Cox regression analysis. The prognostic value for survival in the discovery and verification cohorts was analyzed using Kaplan–Meier survival analyses and receiver operating characteristic (ROC) curves. p < 0.05 was defined as the threshold for significance in Kaplan survival analyses, while p < 0.05 and the area under the curve (AUC) being ≥0.65 were set as cutoffs for ROC curve analyses.

### Tissue collection and immunohistochemical (IHC) analysis

Written informed consent was obtained from all patients enrolled in the study. A total of 44 OS tissues were collected from the Affiliated Hospital of Guizhou Medical University (Guiyang, China), with the approval of the Human Ethics Committee of Guizhou Medical University. Of the 44 OS tissues, 18 tissues were obtained from patients with metastasis at diagnosis, while 26 tissues were obtained from patients without metastasis at diagnosis. These OS tissues were sliced into 4-μm sections and embedded in paraffin, prior to performing IHC experiments. The paraffin-embedded slices were dried at 60°C, deparaffinized by xylene, and soaked in 100%, 90%, 80%, and 70% ethyl alcohol for 10 min (in that order). Antigen retrieval was performed at 120°C in a citrate buffer (pH 6.0) for 10 minutes. After washing with PBS twice, the slices were incubated with 0.3% H_2_O_2_ and 5% bovine serum albumin reagent (Thermo Fisher Scientific, USA) for 30 min, to prevent subsequent non-specific binding. The primary antibodies used were AOC3 (1:500; Cat no. 66834-1-Ig, Proteintech, Wuhan, China), CDK6 (1:200; Cat no. 14052-1-AP, Proteintech, Wuhan, China), COL22A1 (1:250; Cat no. ab121846; Abcam, USA), and RNASE6 (1:100; Cat. ab121111; Abcam, USA), for 14 hours at 4°C. After washing twice with PBS, secondary antibodies were added and incubated for 2 h at room temperature (20°C). Finally, DAB reagent was used to visualize the antigen-antibody complex. The IHC score was determined by the product of the staining area (≤5%, 0; 6%–25%, 1; 26%–50%, 2; 51%–75%, 3; >75%, 4) and depth (none, 0; slight, 1; moderate, 2; strong, 3).

### Construction of the nomogram

A nomogram is a way to visualize the results of logistic or Cox regression analyses. According to the size of the regression coefficient of all independent variables to develop a scoring standard, each value level of each independent variable is given a score. For each patient, a total score can be calculated, and the probability of the outcome time of each patient can then be calculated by the conversion function between the score and probability of the outcome (14). Information on age, sex, risk score, and metastasis status were imported and used to perform univariate and multivariate COX analyses, where these analyses were then used to construct the nomogram. The efficiency of the nomogram was set to 1 year, 3 years, and 5 years.

### Tumor immune dysfunction and exclusion (TIDE) analysis

The TIDE algorithm, developed by Jiang *et al.*, is a computational framework developed to evaluate the potential of tumor immune escape from the gene expression profiles of cancer samples (15). Therefore, the gene expression of OS tissues was downloaded into the TIDE online database (http://tide.dfci.harvard.edu/) to calculate various immune parameters, including microsatellite instability (MSI) score, PDL1 expression, and myeloid-derived suppressor cell (MDSC) levels, and to predict the response rate of immune checkpoint blockade (ICB). The differences in MSI scores between high- and low-risk scores were analyzed using unpaired *t*-tests, while the relationship between PDL1 expression, MDSC levels, and risk scores was analyzed by Pearson co-expression analyses. p < 0.05 was set as the level of significance.

### OncoPredict for drug sensitivity analysis

The OncoPredict R package was developed by Maeser et al. (16) to predict *in vivo* drug responses in cancer patients. OncoPredict fits the gene expression profile of tissues to the half-maximal inhibitory concentration (IC50) of the cancer cell lines to drugs from Genomics of Drug Sensitivity in Cancer (GDSC; https://www.cancerrxgene.org/) and the gene expression profile of cancer lines from the Broad Institute Cancer Cell Line Encyclopedia (CCLE; https://portals.broadinstitute.org/ccle_legacy/home). A total of 198 drugs were calculated, and the sensitivity of the drugs (between the high- and low-risk groups) was analyzed using unpaired *t*-tests. p < 0.05 was set as the threshold for significance.

## Results

### High infiltration of resting dendritic cells was related to poorer prognoses in OS

**Figure 1:**
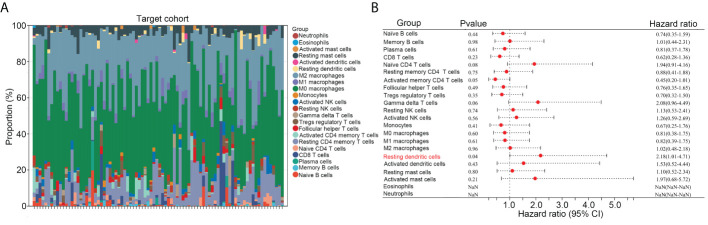
High infiltration of resting dendritic cells was related to poorer prognoses in OS. **(A)** The gene expression matrix of osteosarcoma tissues in TARGET was transformed into expression levels of 22 immune cells, through CIBERSORT. **(B)** The effects of the 22 immune cells on the survival rate of OS were analyzed *via* Kaplan survival analysis.

Previous studies have indicated that dysregulated infiltration of immune cells is associated with the prognosis of patients with OS. We first transformed the gene expression matrix of osteosarcoma tissues in TARGET into the expression levels of 22 types of immune cells, using CIBERSORT (**Figure 1A**). We found that, among the 22 types of immune cells, a high infiltration of resting dendritic cells was associated with a poorer prognosis in patients with OS (HR = 2.18, 95% confidence interval [CI] = 1.01–4.71; **Figure 1B**).

### Genetic characterization of OS tissues with high infiltration of resting dendritic cells

**Figure 2:**
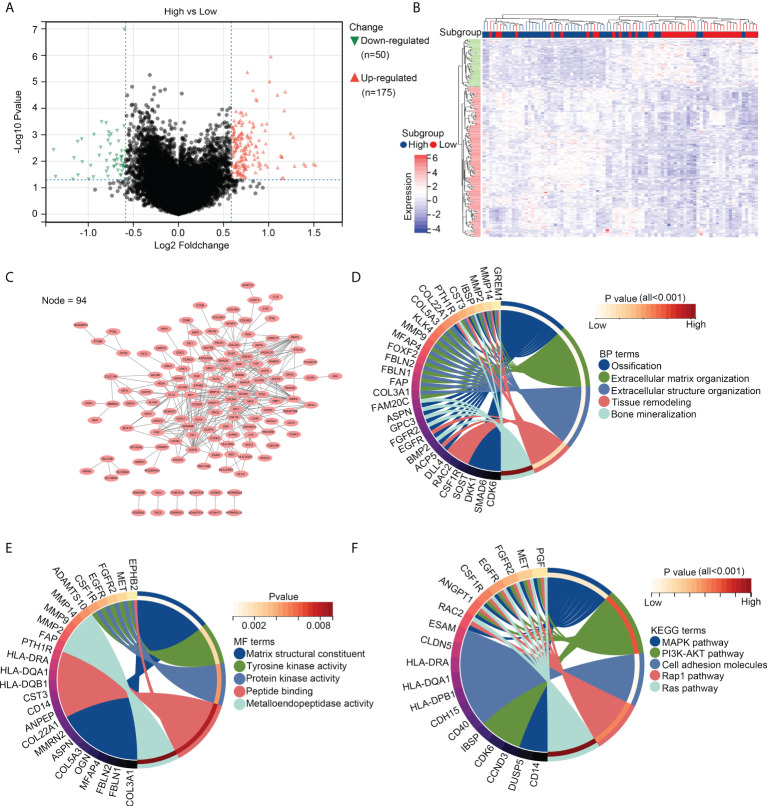
Genetic characterization of OS tissues with high infiltration of resting dendritic cells. **(A)** Volcano plot exhibiting the upregulated and downregulated genes in OS tissues between groups with high and low infiltration of resting dendritic cells. **(B)** Heatmap plot exhibiting the upregulated and downregulated genes in OS tissues between groups with high and low infiltration of resting dendritic cells. **(C)** PPI network indicating the relationship between the 94 genes associated with the infiltration of resting dendritic cells. **(D)** Biological process analyses for the 94 genes associated with the infiltration of resting dendritic cells. **(E)** Molecular function analyses for the 94 genes associated with the infiltration of resting dendritic cells. **(F)** KEGG analysis for the 94 genes associated with the infiltration of resting dendritic cells.

We then divided the OS tissues into high- and low-infiltration groups, according to the median levels of resting dendritic cells within these tissues. DEG analysis was performed, and a total of 175 upregulated genes and 50 downregulated genes were observed in OS tissues with a high infiltration of resting dendritic cells versus those with a low infiltration of resting dendritic cells (**Figures 2A, B**). We then performed a PPI network analysis and found that 94 of these genes were related to others (**Figure 2C**). Therefore, these 94 genes were set as resting dendritic cell-associated genes, and we focused on them. GO analysis revealed that these 94 genes were enriched in “ossification” (BP term; **Figure 2D**), “extracellular matrix organization” (BP term; **Figure 2D**), “extracellular structure organization” (BP term; **Figure 2D**), “tissue remodeling” (BP term; **Figure 2D**), “bone mineralization” (BP term; **Figure 2D**), “matrix structural constituent” (MF term; **Figure 2E**), “tyrosine kinase activity” (MF term; **Figure 2E**), “protein kinase activity” (MF term; **Figure 2E**), “peptide binding” (MF term; **Figure 2E**), and “metalloendopeptidase activity” (MF term; **Figure 2E**). KEGG analysis indicated that these genes were enriched in the MAPK, PI3K-AKT, cell adhesion, Rap1, and Ras pathways (**Figure 2F**).

### Construction of risk model using resting dendritic cell-associated genes

**Figure 3:**
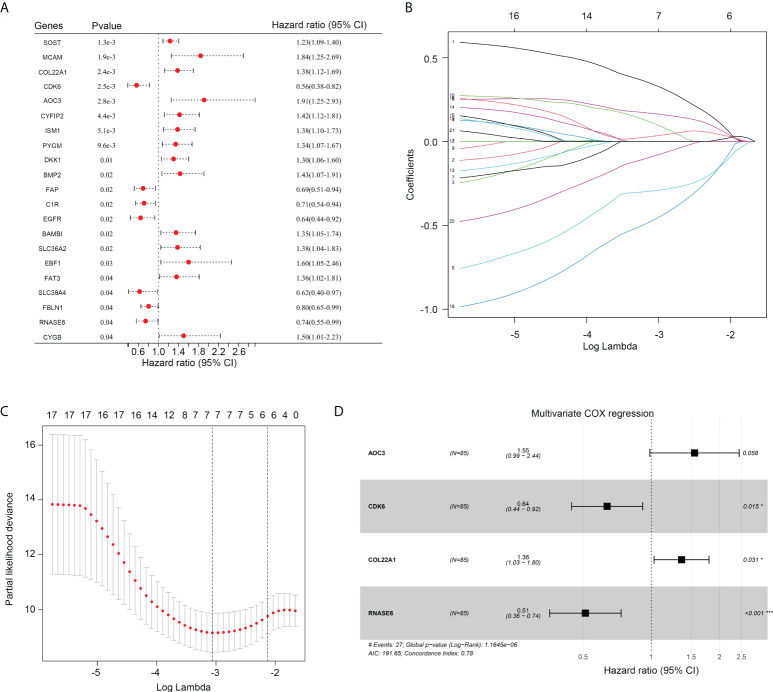
Construction of a risk model. **(A)** Univariate COX regression analysis for the 94 genes associated with the infiltration of resting dendritic cells. **(B, C)** LASSO analysis for other important genes associated with the survival rate of OS. **(D)** The HR and p value of genes (including AOC3, CDK6, COL22A1 and RNASE6) under multivariate COX analysis are shown.

First, univariate COX analyses were performed for these 94 resting dendritic cell-associated genes to calculate their prognostic value. The expression of 14 genes (SOST, MCAM, COL22A1, AOC3, CYFIP2, ISM1, PYGM, DKK1, BMP2, BAMBI, SCL36A2, EBF1, FAT3, and CYGB) was associated with a shorter overall survival rate of OS, while the expression of seven genes (CDK6, FAP, C1R, EGFR, SLC38A4, FBLN1, and RNASE6) was associated with an increased overall survival rate of OS (**Figure 3A**). LASSO COX analysis was then conducted, and seven genes of them (including AOC3, CDK6, COL22A1, EBF1, MCAM, RNASE6, and SLC38A4) were obtained as more important genes (**Figures 3B, C**). Moreover, by performing multivariate COX analyses for these seven more important genes, four genes (AOC3, CDK6, COL22A1, and RNASE6) were used to construct the risk model (risk score = 0.307745*COL22A1 expression + 0.43972*AOC3 expression – 0.44907*CDK6 expression – 0.67038*RNASE6 expression; **Figure 3D**). CDK6, COL22A1, and RNASE6 also had prognostic value in TARGET patients with OS, as per multivariate COX analyses (**Figure 3D**).

### The risk model exhibited high prognostic value in the TARGET discovery cohort

**Figure 4:**
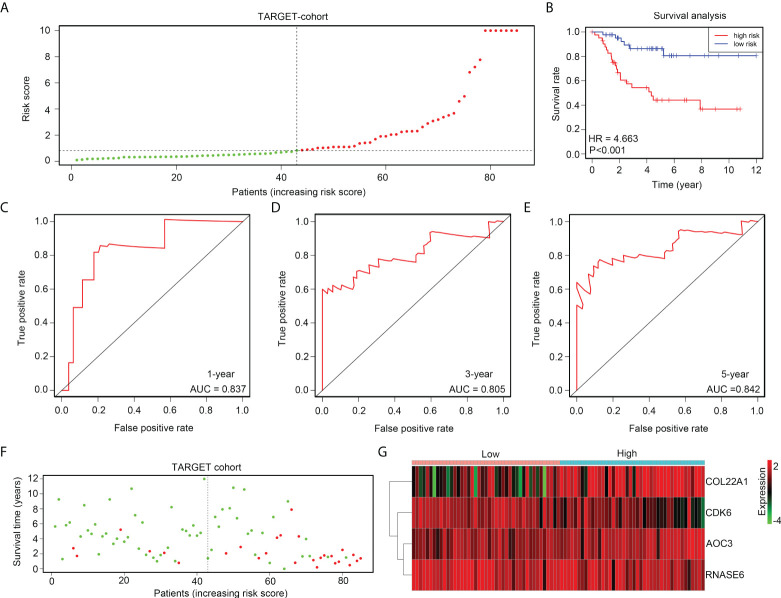
The risk model exhibits high prognostic value in the TARGET discovery cohort. **(A)** OS tissues in TARGET were divided into low- and high-risk groups, according to median risk scores. **(B)** Kaplan survival analysis indicated the difference between low- and high-risk group OS tissues in TARGET. **(C–E)** ROC analyses of the risk model for the 1-year, 3-year, and 5-year survival rates for OS patients in TARGET. **(F)** Alive and death cases between low- and high-risk group OS tissues in TARGET. **(G)** Expression of COL22A1, CDK6, RNASE6 and AOC3 between low- and high-risk group OS tissues in TARGET.

The prognostic value of the risk model was first determined in the TARGET discovery cohort. Therefore, OS tissues in TARGET were divided into high- and low-risk groups, according to median risk scores (**Figure 4A**). A shorter overall survival rate was observed in the high-risk group than in the low-risk group (**Figure 4B**). ROC analysis demonstrated that the diagnostic value (AUC) of this risk model for the 1-year, 3-year, and 5-year survival rates of patients with OS in the TARGET cohort were 0.837, 0.805, and 0.842, respectively (**Figures 4C–E**). Moreover, high-risk groups had a higher proportion of deaths (**Figure 4F**). Furthermore, we found that the expression of COL22A1 and AOC3 was increased in high-risk score groups, whereas the expression of CDK6 and RNASE6 was reduced in high-risk score groups (**Figure 4G**). Taken together, these results indicate that the risk model exhibited a high prognostic value in the TARGET discovery cohort.

### The risk model exhibited high prognostic value in the GSE21257 verification cohort

**Figure 5:**
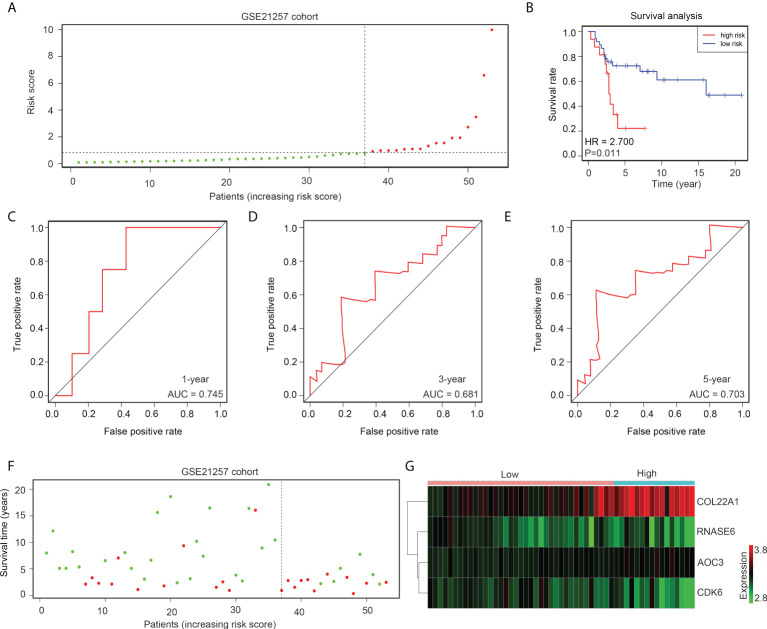
The risk model exhibits high prognostic value in the GSE21257 verification cohort. **(A)** OS tissues in GSE21257 were divided into low- and high-risk groups according to median risk scores. **(B)** Kaplan survival analysis indicated the difference between low- and high-risk group OS tissues in GSE21257. **(C–E)** ROC analysis of the risk model for the 1-year, 3-year, and 5-year survival rates for OS patients in GSE21257. **(F)** Alive and death cases between low- and high-risk group OS tissues in GSE21257. **(G)** Expression of COL22A1, CDK6, RNASE6 and AOC3 between low- and high-risk group OS tissues in GSE21257.

The gene expression profile of GSE21257 was set as the verification cohort, and the tissues were divided into high- and low-risk groups according to the medium-risk score (**Figure 5A**). The results indicated that a lower overall survival rate was observed in the high-risk group than in the low-risk group (**Figure 5B**). ROC analysis demonstrated that the diagnostic value (AUC) of this risk model for the 1-year, 3-year, and 5-year survival rates of patients with OS in GSE21257 were 0.745, 0.681, and 0.703, respectively (**Figures 5C–E**). Moreover, the results indicated that the high-risk groups also had a higher proportion of deaths (**Figure 5F**). Furthermore, we found that the expression of COL22A1 and AOC3 was also elevated in the high-risk group, whereas CDK6 and RNASE6 expression was decreased in the high-risk group (**Figure 5G**). In conclusion, the risk model exhibited high prognostic value in the verification cohort GSE21257.

### The risk model had the potential to predict metastasis in patients with OS

**Figure 6:**
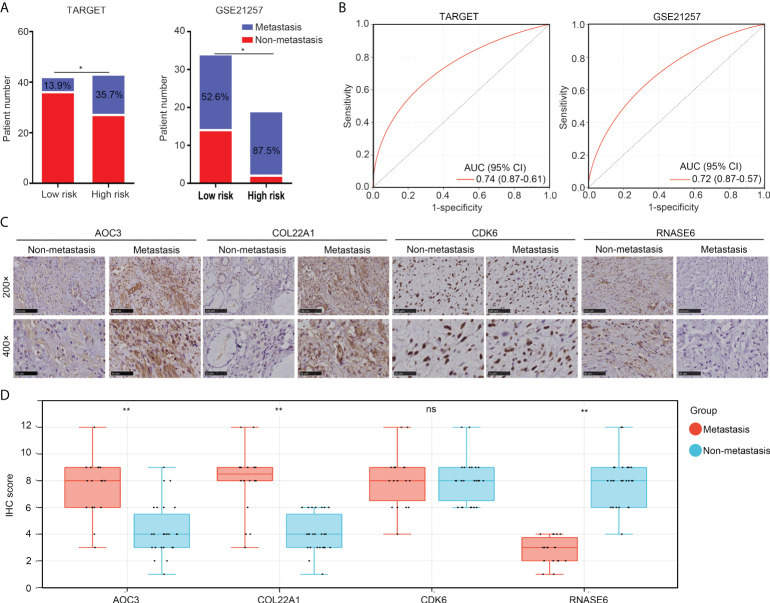
The risk model has the potential to predict metastasis in patients with OS. **(A)** Non-metastasis and metastasis cases between low- and high-risk group OS tissues in the TARGET and GSE21257 cohorts. **(B)** ROC analysis for the diagnostic value of the risk model in the prediction of OS tissue metastasis. **(C, D)** IHC was performed to detect the expression of COL22A1, CDK6, RNASE6 and AOC3 in non-metastasis and metastasis OS tissues (magnification 200× and 400×). *p < 0.05; **p < 0.01; ns, no significant.

More metastasis cases (TARGET, 35.7% and GSE21257, 87.5%) were found in the high-risk group than in the low-risk group (TARGET, 13.9% and GSE21257, 52.6%; **Figure 6A**). ROC analyses indicated that the diagnostic values (AUC) of the risk model for predicting metastasis were 0.741 and 0.720 for OS patients in TARGET and GSE21257, respectively (**Figure 6B**). Moreover, we detected the expression of AOC3, COL22A1, CDK6, and RNASE6 in OS tissues obtained from patients with metastasis (n = 18) versus those without metastasis (n = 26), using IHC. We found that the protein levels of AOC3 and COL22A1 were increased, and RNASE6 was decreased in OS tissues from patients with metastasis (**Figures 6C, D**). These results indicate that the risk model has the potential to predict metastasis in patients with OS.

### The risk model can act as an independent factor for predicting OS patient prognosis

**Figure 7:**
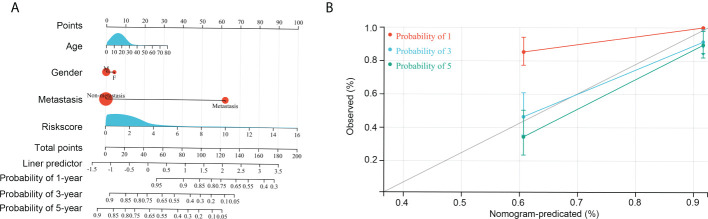
Construction of the nomogram. **(A)** Age, gender, risk score, and metastasis was used to construct the nomogram. **(B)** Efficiency of the nomogram in the 1-year, 3-year, and 5-year survival rates of patients with OS.

Information on age, sex, metastasis status, and risk score of all patients with OS in the TARGET and GSE21257 groups was used to conduct Cox regression analyses. Risk score and metastasis status could act as independent factors for predicting OS patient prognosis (**Table 1**). To further help in predicting OS patient prognosis, a nomogram was constructed (**Figure 7A**), which showed high prognostic value for the 1-year, 3-year, and 5-year survival rates (**Figure 7B**).

**Table 1:** Univariate and multivariate COX regression analyses for age, gender, risk score, and metastasis status in OS tissues.

Characteristics	Total(N)	HR(95% CI) Univariate analysis	P value Univariate analysis	HR(95% CI) Multivariate analysis	P value Multivariate analysis
Age	138	1.009 (0.979-1.040)	0.566	–	–
Gender	138	–	0.955	–	–
Female	56	Reference	–	–	–
Male	82	0.970 (0.545-1.727)	0.955	–	–
Metastasis	138	–	<0.001	–	–
Yes	56	Reference	–	–	–
No	82	0.173 (0.093-0.323)	<0.001	0.200 (0.105-0.379)	<0.001
riskScore	138	1.223 (1.146-1.305)	<0.001	1.172 (1.099-1.250)	<0.001

### The high-risk group patients with OS exhibited resistance to ICB

**Figure 8:**
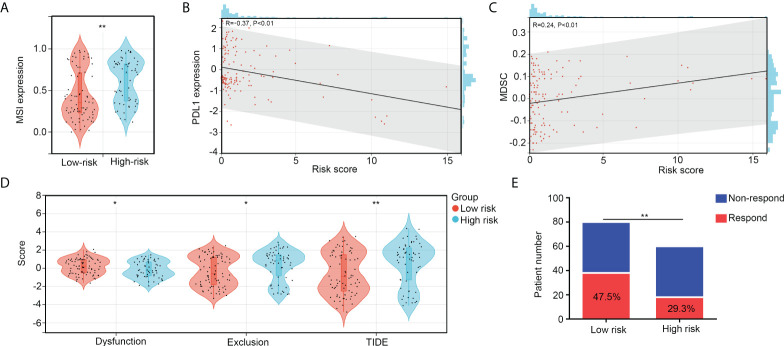
The high-risk group patients with OS exhibit resistance to ICB. **(A)** MSI scores between high- and low-risk group OS tissues. **(B)** Co-expression relationships between risk scores and PDL1 expression. **(C)** Co-expression relationships between risk scores and MDSC levels. **(D)** The difference of exclusion, dysregulation, and TIDE scores between high- and low-risk group OS tissues. **(E)** Predication of non-responder and responder numbers after ICB in high- and low-risk group OS tissues. *p < 0.05, **p < 0.01.

We then analyzed immunological characteristics of the high- and low-risk OS tissues. We found that microsatellite instability (MSI) scores were higher in the high-risk group than in the low-risk group (**Figure 8A**). The risk score was negatively associated with PDL1 expression (R = -0.37, p < 0.01; **Figure 8B**) and positively associated with MDSC cell levels (R = 0.24, p < 0.01; **Figure 8C**). Moreover, by performing TIDE analyses, we found that both exclusion and TIDE scores were higher in the high-risk groups of patients with OS than in the low-risk groups, while dysregulation scores were reduced in the high-risk groups (**Figure 8D**). Moreover, the proportion of non-responders to ICB therapy was higher in the high-risk group (70.7%) than in low risk group (52.5%; **Figure 8E**). These results indicate that the high-risk group of patients with OS exhibited resistance to ICB.

### Selecting suitable drugs for the high-risk group of patients with OS, *via* OncoPredict

**Figure 9:**
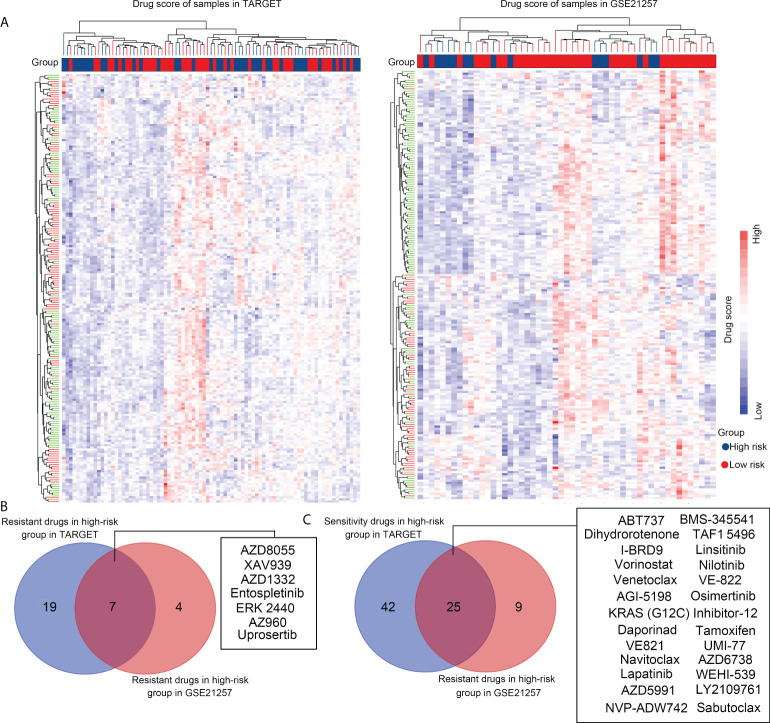
Selecting suitable drugs for high-risk group patients with OS *via* OncoPredict. **(A)** OncoPredict was used to transform the gene expression profile of OS tissues in the TARGET and GSE21257 groups into a drug sensitivity matrix of 198 drugs. **(B)** Resistant drugs identified in high-risk groups in both the TARGET and GSE21257 cohorts. **(C)** Sensitive drugs identified in high-risk groups in the TARGET and GSE21257 cohorts.

To explore suitable drugs for patients with high-risk scores, we transformed the gene expression of OS tissues in the TARGET and GSE21257 groups into a drug sensitivity matrix, using the OncoPredict algorithm (**Figure 9A**). All scores for each sample are exhibited in **Supplementary Table 1**. OS tissues from high-risk group patients exhibited greater resistance to seven drugs, including those of AZD8055 (targeting drug, mTOR inhibitor), XAV939 (targeting drug, tankyrase inhibitor), AZD1332 (targeting drug, receptor tyrosine kinase inhibitor), Entospletinib (targeting drug, Syk inhibitor), ERK 2440 (targeting drug, ERK inhibitor), AZ960 (targeting drug, JAK inhibitor), and Uprosertib (targeting drug, AKT inhibitor), than those from low-risk group patients (**Figure 9B**). OS tissues from high-risk group patients were more sensitive to 25 drugs, including those of ABT737 (targeting drug, Bcl-2 inhibitor), BMS-345541 (targeting drug, IKK inhibitor), Navitoclax (targeting drug, Bcl-2 inhibitor), TAF1 5496 (targeting drug, TAF1 inhibitor), I-BRD9 (targeting drug, BRD9 inhibitor), Linsitinib (targeting drug, IGF-1R inhibitor), Vorinostat (targeting drug, HDAC inhibitor), Nilotinib (targeting drug, Bcr-abl inhibitor), Venetoclax (targeting drug, Bcl-2 inhibitor), VE-822 (targeting drug, ATM inhibitor), AGI-5198 (targeting drug, IDH inhibitor), Osimertinib (targeting drug, EGFR inhibitor), Daporinad (targeting drug, NMPRTase inhibitor), Tamoxifen (Chemotherapy drug), VE821 (targeting drug, ATM inhibitor), UMI-77 (targeting drug, Bcl-2 inhibitor), Dihydrorotenone (mitochondrial inhibitor), KRAS (G12C) Inhibitor-12 (targeting drug, KRAS inhibitor), AZD6738 (targeting drug, ATR inhibitor), WEHI-539 (targeting drug, BCL-XL inhibitor), Sabutoclax (targeting drug, Bcl-2 inhibitor), Lapatinib (targeting drug, EGFR/HER2 inhibitor), AZD5991 (targeting drug, MCL-1 inhibitor), LY2109761 (targeting drug, TGF-β Receptor I/II inhibitor) and NVP-ADW742 (targeting drug, IGF1R inhibitor; **Figure 9C**) than were OS tissues from low-risk group patients. We believe that these drugs may help in the treatment of OS patients with high-risk scores.

## Discussion

The effectiveness of immunotherapy in the treatment of several cancers has gained recognition in recent years. Similarly, immunotherapy is expected to be widely used in the treatment of OS. However, compared with its success in preclinical studies, the clinical effectiveness of immunotherapy is limited by different immune microenvironments in OS tissues (17). For example, Groisberget et al. demonstrated that only 26% of patients with OS yielded a partial response or experienced stable disease progression after immunotherapy (18). Regarding clinical traits assessed by Ullenhag et al., the effective rate was 30% (19). Therefore, the identification of genes associated with the immune characteristics of OS may contribute to improved diagnosis of and therapy for OS.

In the present study, we first calculated the number of immune cells in OS tissues. We found that high levels of resting dendritic cells were associated with poorer prognoses. Being the most typical type of antigen-presenting cell, dendritic cells bridge the gap between innate and adaptive immunity, which also includes antitumor T-cell activation. Dendritic cells are activated during immunoreaction. Activated dendritic cells recognize and process immune signals and present antigens to T cells, thus activating immunological cascades (20, 21). Therefore, high levels of resting dendritic cells indicate lower levels of immunoreaction. Consistent with previous studies, our results indicate that activated dendritic cells may contribute to improving OS survival rate.

We then analyzed gene expression differences between the high and low dendritic cell group OS tissues. A total of 94 key dendritic cell-associated genes were identified, and four genes associated with the survival of patients with OS (including AOC3, CDK6, COL22A1, and RNASE6) were used to construct the risk model. AOC3 encodes a cell adhesion protein that mediates lymphocyte binding to peripheral lymph node vascular endothelial cells during lymphocyte extravasation and recirculation, in an L-selectin-independent fashion (22). AOC3 is dysregulated in various cancers, with contradictory roles. In colorectal cancer, AOC3 expression is reduced in both *in situ* tissues and serum, and reduced AOC3 expression is related to poorer prognoses (23). In breast cancer, AOC3 is highly expressed and is positively associated with lymphatic invasion and distant metastasis (24). CDK6 is a serine/threonine-protein kinase involved in the control of the cell cycle and cell differentiation, and has the potential to promote G1/S transition (25). Oncogenic effects have been widely reported in various cancer types, including those of OS. COL22A1 encodes a collagen family member that is thought to be involved in stabilizing myotendinous junctions and strengthening skeletal muscle attachment (26). High expression of COL22A1 was observed in head and neck cancer, and was correlated with a decrease in disease-free survival (27). RNASE6 is a secreted protein with broad-spectrum antimicrobial activity against pathogenic bacteria (28). However, the role of RNASE6 in cancer is unclear. In the present study, we found that the risk model constructed by AOC3, CDK6, COL22A1, and RNASE6 showed distinct prognostic value for OS in both TARGET and GSE21257 groups, as well as for predicting metastasis. Furthermore, this risk model was found to be an independent factor for OS. We believe that this risk model may aid in OS diagnosis.

Immunological characteristics, including tumor mutation burden (TMB) and the MSI score of tumor tissues, can indicate the therapeutic effects of ICB (29, 30). Previous studies have indicated that high TMB and MSI in tumor tissues indicate beneficial effects after ICB (31). To analyze the benefit for patients with OS, we analyzed the MSI score in the high- and low-risk groups. Our results indicated that the MSI score was higher in the high-risk group, suggesting that the high-risk group patients had a greater benefit from ICB. However, after calculating other parameters, we found that the risk score was negatively associated with PDL1 expression and positively associated with MDSC levels. High exclusion and TIDE scores were observed in the OS tissues of the high-risk group, while the dysregulation score was reduced. These parameters inversely indicated that the high-risk group had a lesser benefit from ICB, while the low-risk group had a greater benefit from ICB. Combining these parameters, we speculated that, although high MSI scores would induce immune responses, the lack of activated dendritic cells ultimately prevents T cells from being activated to effectively kill tumors. Similarly, because this type of immune escape was not due to PDL1 overexpression in OS tumors in the high-risk group, some ICB strategies for targeting surface antigens of T cells were less beneficial. This supposition was consistent with some evidence from clinical traits, where supplementation of activated dendritic cells combined with ICB was more beneficial than ICB alone, in the context of OS (32, 33). Based on this evidence, we consider that the risk model provided in the present study has remarkable value in guiding ICB. Finally, we performed OncoPredict and found that the high-risk group OS tissues were resistant to seven drugs and sensitive to 25 drugs. This evidence may also contribute to guiding chemotherapy and targeted therapies for OS.

Our study has some limitations. First, whether and how the four genes (AOC3, COL22A1, CDK6, and RNASE6) affect the activation of dendritic cells has not been studied. The sensitivity of OS tissues to chemotherapy and targeted drugs also needs to be verified.

In conclusion, the signature constructed by four key genes associated with the level of dendritic cells (AOC3, COL22A1, CDK6, and RNASE6) had remarkable prognostic value for predicting prognosis and metastasis in patients with OS, as well as guiding ICB, chemotherapy, and targeted chemotherapy for OS.

## Data availability statement

The datasets presented in this study can be found in online repositories. The names of the repository/repositories and accession number(s) can be found in the article/**Supplementary Material**.

## Ethics statement

Written informed consent was obtained from the individual(s), and minor(s)’ legal guardian/next of kin, for the publication of any potentially identifiable images or data included in this article.

## Author contributions

CH, HC, and XT designed the experiments in this study. RP, FP, and ZZ performed the analyses and experiments. SL, YSY, and YY performed these experiments. All the authors have read and agreed to submit the final version of the manuscript.

## Funding

The present study was funded by the National Natural Science Foundation of China (82060491, 82160665 and 82160543), the Guiyang High-level Innovative Youth Health Talents Training Program Project (2020 Zhuweijian Technology Contract No. 018), the Department of Science and Technology of Guizhou ([2022]232), the Basic Research Program of the Guizhou Province Technology Bureau (No. ZK [2021] General-568), the Family Planning Commission of Guizhou Province (gzwkj2021-442), and The 2021 National Foundation Cultivation Program of Guizhou Medical University (20NSP080).

## Conflict of interest

The authors declare that the research was conducted in the absence of any commercial or financial relationships that could be construed as a potential conflict of interest.

## Publisher’s note

All claims expressed in this article are solely those of the authors and do not necessarily represent those of their affiliated organizations, or those of the publisher, the editors and the reviewers. Any product that may be evaluated in this article, or claim that may be made by its manufacturer, is not guaranteed or endorsed by the publisher.

## Supplementary material

Supplementary Table 1 Drug score of 198 drugs in each sample.
